# Travelling wave solutions in a negative nonlinear diffusion–reaction model

**DOI:** 10.1007/s00285-020-01547-1

**Published:** 2020-11-20

**Authors:** Yifei Li, Peter van Heijster, Robert Marangell, Matthew J. Simpson

**Affiliations:** 1 School of Mathematical Sciences, Queensland University of Technology, Brisbane, QLD Australia; 2 Biometris, Wageningen University and Research, Wageningen, The Netherlands; 3 School of Mathematics and Statistics, University of Sydney, Sydney, NSW Australia

**Keywords:** Nonlinear diffusion, Travelling wave solutions, Geometric methods, Phase plane analysis, Spectral stability, 92C17, 92D25, 35K57, 35B35

## Abstract

We use a geometric approach to prove the existence of smooth travelling wave solutions of a nonlinear diffusion–reaction equation with logistic kinetics and a convex nonlinear diffusivity function which changes sign twice in our domain of interest. We determine the minimum wave speed, $c^*$, and investigate its relation to the spectral stability of a desingularised linear operator associated with the travelling wave solutions.

## Introduction

Invasion processes have been studied with mathematical models, especially partial differential equations (PDEs), for many years; see, for example, Murray (2002) and references therein. These models describe, for instance, how cells are transported to new areas in which they persist, proliferate, and spread (Mack et al. 2000). To incorporate information about individual-level behaviours in invasion processes, lattice-based discrete models are widely used (Deroulers et al. 2009; Johnston et al. 2017, 2012; Simpson et al. 2010c). In these discrete models, individual agents are permitted to move, proliferate and die on a lattice, and the average density of agents is related to PDE descriptions obtained using truncated Taylor series in the continuum limit (Anguige and Schmeiser 2009; Codling et al. 2008). The macroscopic behaviour described by the PDEs in terms of expected agent density reflects the individual microscopic behaviour. Travelling wave solutions are of particular interest among the macroscopic behaviours arising from these continuum models, as they reflect various modes of microscopic invasive behaviours. One famous model exhibiting travelling wave solutions is the Fisher–KPP equation (KPP refers to Kolmogorov, Petrovsky, Piskunov) proposed in 1937 to study population dynamics with linear diffusion and logistic growth (Fisher 1937; Kolmogorov et al. 1937). The existence and stability of travelling wave solutions of the Fisher–KPP equation has been widely studied, see, for instance, Aronson and Weinberger (1978), Fisher (1937), Harley et al. (2015), Kolmogorov et al. (1937), Larson (1978), Murray (2002) and Sherratt (1998).

The Fisher–KPP equation can be derived as a continuum limit of a discrete model under the assumption that the population of cells can be treated as a uniform population without any differences in subpopulations (Bramson et al. 1986). However, differences between individual and collective behaviour have been observed in cell biology and ecology in practice. For instance, in cell biology, isolated cells called *leader cells* are more motile than the grouped cells, called *follower cells* (Poujade et al. 2007). Also, contact interactions lead to different motility rates between isolated cells and grouped cells in the migration of breast cancer cells (Simpson et al. 2010c, 2014), glioma cells (Khain et al. 2011), would healing processes (Khain et al. 2007) and the development of the enteric nervous system (Druckenbrod and Epstein 2007). In ecology, the population growth rate of some species decreases as their populations reach small sizes or low densities (Courchamp et al. 1999). This phenomenon is usually referred to as the Allee effect (Allee and Bowen 1932).

To describe the invasion process and reflect the difference between collective and individual behaviour, Johnston and coworkers introduced a discrete model considering birth, death and movement events of agents that are isolated or grouped on a simple one-dimensional lattice (Johnston et al. 2017). A discrete conservation statement describing $\delta U_{j}$, which is the change of the occupancy of a lattice site *j* during a time step $\tau$, gives

1 $$\begin{aligned} \delta U_j= & {} \frac{P^i_m}{2}[U_{j-1}(1-U_j)(1-U_{j-2})+U_{j+1}(1-U_j)(1-U_{j+2})\nonumber \\&-2U_{j}(1-U_{j-1})(1-U_{j+1})]\nonumber \\&+\frac{P^g_m}{2}[U_{j-1}(1-U_j)+U_{j+1}(1-U_j)-U_{j}(1-U_{j-1})-U_{j}(1-U_{j+1})]\nonumber \\&-\frac{P^g_m}{2}[U_{j-1}(1-U_j)(1-U_{j-2})+U_{j+1}(1-U_j)(1-U_{j+2})\nonumber \\&-2U_{j}(1-U_{j-1})(1-U_{j+1})]\nonumber \\&+\frac{P^i_p}{2}[U_{j-1}(1-U_j)(1-U_{j-2})+U_{j+1}(1-U_j)(1-U_{j+2})]\nonumber \\&+\frac{P^g_p}{2}[U_{j-1}(1-U_j)+U_{j+1}(1-U_j)]\nonumber \\&-\frac{P^g_p}{2}[U_{j-1}(1-U_j)(1-U_{j-2})+U_{j+1}(1-U_j)(1-U_{j+2})]\nonumber \\&-P^i_d[U_j(1-U_{j-1})(1-U_{j+1})]-P^g_dU_j+P^g_d[U_j(1-U_{j-1})(1-U_{j+1})].\nonumber \\ \end{aligned}$$

**Fig. 1:**
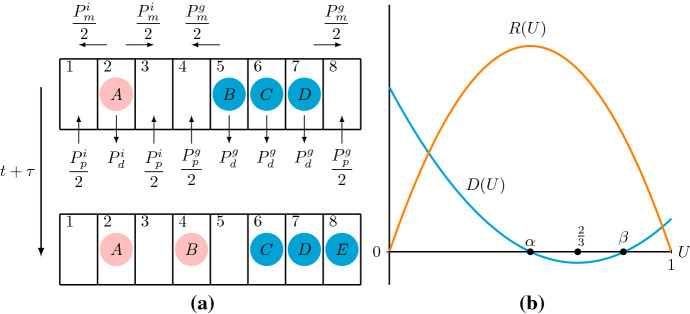
**a** One possible time step of the lattice-based discrete model of Johnston et al. (2017): a new grouped agent (agent E) is born and the grouped agent B moves from lattice site 5 to lattice site 4 to become an isolated agent. Pink circles represent isolated agents with birth rate $P^i_p$, death rate $P^i_d$ and motility rate related to $P^i_m$; cyan circles represent grouped agents with birth rate $P^g_p$, death rate $P^g_d$ and motility rate $P^g_m$. **b** presents a diffusivity function *D*(*U*), given by (3) (cyan curve) satisfying $D_i>4D_g$ which makes *D*(*U*) change sign twice on (0, 1), and the kinetic term *R*(*U*), given by (5) (orange curve) which is positive on (0, 1) and zero at end points $U=0$ and $U=1$ (colour figure online)

Here, $U_j$ represents the probability that an agent occupies lattice site *j*, thus, $1-U_j$ represents the probability that lattice site *j* is vacant (Simpson et al. 2010a). $P^i_m$ and $P^g_m$ represents the probability per time step that isolated or grouped agents, respectively, attempt to step to a nearest neighbour lattice site; $P^i_p$ and $P^g_p$ represents the probability per time step that isolated or grouped agents, respectively, attempt to undergo a proliferation event and deposit a daughter agent at a nearest neighbour lattice site; $P_d^i$ and $P_d^g$ represents the probability per time step that isolated or grouped agents, respectively, die, and are removed from the lattice. See Fig. 1a for a schematic of the lattice-based discrete model.

To obtain a continuous description, Johnston and coworkers treat $U_j$ as a continuous function, *U*(*x*, *t*), and divide (1) by the time step $\tau$. Next, they expand all terms in (1) in a Taylor series around $x=j\varDelta$, where $\varDelta$ is the lattice spacing, and neglect terms of $\mathcal {O}(\varDelta ^3)$ (Simpson et al. 2010a). As $\varDelta \rightarrow 0$ and $\tau \rightarrow 0$ with the ratio ${\varDelta ^2}/{\tau }$ held constant (Codling et al. 2008; Simpson et al. 2010a), they obtain a nonlinear diffusion–reaction equation

2 $$\begin{aligned} \frac{\partial U}{\partial t}=\frac{\partial }{\partial x}\left( D(U)\frac{\partial U}{\partial x}\right) +{R}\left( U\right) , \end{aligned}$$

where

3 $$\begin{aligned} D\left( U\right) =D_i\left( 1-4U+3U^2\right) +D_g\left( 4U-3U^2\right) , \end{aligned}$$

is the nonlinear diffusivity function, and

4 $$\begin{aligned} R\left( U\right) =\lambda _g U\left( 1-U\right) +\left( \lambda _i-\lambda _g-K_i+K_g\right) U\left( 1-U\right) ^2-K_g U, \end{aligned}$$

is the kinetic term. Furthermore, the parameters are given by

$$\begin{aligned} D_g= & {} \lim _{\varDelta ,\tau \rightarrow 0}\frac{P^g_m\varDelta ^2}{2\tau },\quad D_i=\lim _{\varDelta ,\tau \rightarrow 0}\frac{P^i_m\varDelta ^2}{2\tau },\quad \lambda _g=\lim _{\tau \rightarrow 0}\frac{P^g_p}{\tau },\\ \lambda _i= & {} \lim _{\tau \rightarrow 0}\frac{P^i_p}{\tau },\quad K_g=\lim _{\tau \rightarrow 0}\frac{P^g_d}{\tau },\quad K_i=\lim _{\tau \rightarrow 0}\frac{P^i_d}{\tau }, \end{aligned}$$

where we require that $P^i_p,P^g_p,P^i_d,P^g_d$ are $\mathcal {O}(\tau )$ (Simpson et al. 2010a). Here, *U*(*x*, *t*) denotes the total density of the agents at position $x\in \mathbb {R}$ and time $t\in \mathbb {R}_{+}$; $D_i\ge 0$ and $D_g\ge 0$ are diffusivities of the isolated and grouped agents, respectively; $\lambda _i\ge 0$ and $\lambda _g\ge 0$ are the proliferation rates of isolated and grouped agents, respectively; $K_i\ge 0$ and $K_g\ge 0$ are the death rates of isolated and grouped agents, respectively (Johnston et al. 2017). Note that this particular form (2) was proposed by Johnston et al. (2017). This was one of the first studies that proposed a nonlinear diffusion–reaction model to a mean-field description of a lattice-based stochastic model incorporating agent movement, proliferation and death. Previous work leading to nonlinear diffusion equations only considered the movement of agents and thus did not involve kinetic terms (Johnston et al. 2012; Anguige and Schmeiser 2009).

**Fig. 2:**
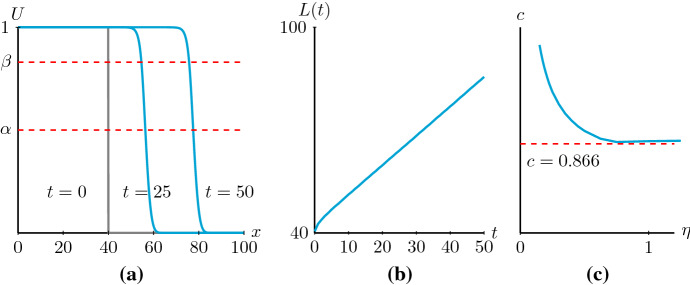
**a** The evolution of a Heaviside initial condition to a smooth travelling wave solution obtained by simulating (2) with (3) and (5) with parameters $D_i=0.25$, $D_g=0.05$ and $\lambda =0.75$. We use a finite difference method with space step $\delta x=0.1$, time step $\delta t=0.01$ and no-flux boundary conditions. Notice that $D(U)=0$ at $\alpha =0.5$ and $\beta \approx 0.83$. **b** The position of the wave *L(t)*, measured by the left-most leading edge point where *U* is smaller than 10–5, indicating that the solution is travelling at a constant speed *c* $=$ 0.864. **c** The wave speed as a function of the initial condition $U(x,0)=1/2+\text {tanh}\left( -\eta (x-40)\right) /2$. Notice that as $\eta$ grows to infinity this initial condition limits to the Heaviside initial condition used for the simulation in (**a**), and the wave speed converges to $c\approx 0.864$. The minimum wave speed $c^*=2\sqrt{\lambda D_i}\approx 0.866$ (11) (colour figure online)

In this manuscript, we study the effect that aggregation, which is modelled with a nonlinear diffusivity function that goes negative (Simpson et al. 2010b), has on the dynamics of the continuous PDE model. Therefore, we assume that $D_i>4D_g$ such that *D*(*U*) given by (3) is convex and changes sign twice in our domain of interest (additionally, see Sect. 4.2 for a short discussion related to the other case). For simplicity, we furthermore assume equal proliferation rates, $\lambda =\lambda _i=\lambda _g$, and no agent death, $K_i=K_g=0$. This way, the kinetic term simplifies to a logistic term

5 $$\begin{aligned} R\left( U\right) =\lambda U\left( 1-U\right) , \end{aligned}$$

and $D\left( U\right)$ has a sign condition:

6 $$\begin{aligned} D\left( U\right) >0 \quad \text {for}\quad U\in \left[ 0,\alpha \right) \cup \left( \beta ,1\right] ,\quad D\left( U\right) <0\quad \text {for} \quad U\in \left( \alpha ,\beta \right) , \end{aligned}$$

where the interval where $D(U)<0$ is centred at $U=2/3$, and $\alpha , \beta$ are given by

7 $$\begin{aligned} \alpha =\frac{2}{3}-\frac{\sqrt{D_{i}^{2}+4D_{g}^2-5D_{i}D_{g}}}{3\left( D_i-D_g\right) },\quad \beta =\frac{2}{3}+\frac{\sqrt{D_{i}^{2}+4D_{g}^2-5D_{i}D_{g}}}{3\left( D_i-D_g\right) }, \end{aligned}$$

with $1/3<\alpha <2/3$ and $2/3<\beta <1$, see Fig. 1b. That is, we have negative diffusion for $U\in (\alpha ,\beta )$. The relation that $D_i$ is larger than $D_g$ indicates that isolated agents are more active than grouped agents, which agrees with the experimental observation that *leader cells* are more motile than *follower cells* (Poujade et al. 2007; Simpson et al. 2014). Ferracuti et al. (2009) showed the existence of travelling wave solutions for a range of positive wave speeds for (2) with general convex *D*(*U*) that changes sign twice on (0, 1) and *R*(*U*) given by (5) based on the *comparison method* introduced by Aronson and Weinberger (1978). Related studies proved the existence of travelling wave solutions for a similar range of speeds for nonlinear diffusion–reaction equations with different *D*(*U*) and different *R*(*U*): Malaguti and Marcelli (2003) studied (2) with a logistic kinetic term and a nonlinear diffusivity function satisfying

$$\begin{aligned} D(0)=0\quad \text {and}\quad D(0)>0\quad \text {for all}\quad U\in (0,1]. \end{aligned}$$

Maini et al. (2006) studied (2) with a logistic kinetic term and a nonlinear diffusivity function satisfying

8 $$\begin{aligned} D(U)>0\quad \text {in}\quad (0,\theta )\quad \text {and}\quad D(U)<0 \quad \text {in}\quad U\in (\theta ,1), \end{aligned}$$

for some given $\theta \in (0,1)$ and with $D(0)=D(\theta )=D(1)=0$. In addition, Maini et al. (2007) studied (2) with (8) and a bistable kinetic term satisfying

$$\begin{aligned} R(0)=R(\phi )=R(1)=0,\quad R(U)<0\quad \text {in} \quad U\in (0,\phi )\quad \text {and}\quad R(U)>0\quad \text {in} \quad U\in (\phi ,1). \end{aligned}$$

A travelling wave solution of (2) is a solution that travels with constant speed $c>0$ and constant wave shape, and that asymptotes to 1 as $x\rightarrow -\infty$ and to 0 as $x\rightarrow \infty$ (i.e. the roots of *R*(*U*)). We only consider positive wave speeds since (2) with (3) and (5) is monostable with a Fisher–KPP imprint, that is, $U\equiv 1$ is a PDE stable solution of (2), while $U\equiv 0$ is a PDE unstable solution (in an appropriate function space which will be introduced in Sect. 3). Hence, to study travelling wave solutions we introduce the travelling wave coordinate $z=x-ct$, where $z\in \mathbb {R}$ and $c>0$, and write (2) in its travelling wave coordinate

9 $$\begin{aligned} \frac{\partial U}{\partial t}=\frac{\partial }{\partial z}\left( D(U)\frac{\partial U}{\partial z}\right) +c\frac{\partial U}{\partial z}+R(U). \end{aligned}$$

A travelling wave solution is now a stationary solution to (9), that is, ${\partial U}/{\partial t}=0$ (Sandstede 2002). In other words, a travelling wave solution is a solution to the second-order ordinary differential equation (ODE)

10 $$\begin{aligned} \frac{d}{d z}\left( D(u)\frac{du}{dz}\right) +c\frac{du}{dz}+R(u)=0, \end{aligned}$$

with asymptotic boundary conditions ${\lim \nolimits _{z\rightarrow -\infty }}u=1$ and ${\lim \nolimits _{z\rightarrow \infty }}u=0$.

In this manuscript, we show the following result:

### Theorem 1

Model (2) with (3) and (5) and $D_i>4D_g$ supports smooth monotone nonnegative travelling wave solutions for

11 $$\begin{aligned} c\ge 2\sqrt{\lambda D_i}=:c^*. \end{aligned}$$

This theorem agrees with the result of Ferracuti et al. (2009), and because of the specific nonlinear diffusivity function, we can further extend their results. Moreover, instead of the comparison method used by Ferracuti et al. (2009), we use a geometric approach to prove the existence of travelling wave solutions. This geometric approach has the advantage that it can also be used to study shock-fronted, discontinuous travelling wave solutions (Wechselberger and Pettet 2010; Harley et al. 2014a, b). While shock-fronted travelling wave solutions are not the focus in this manuscript, we show in the final section that they do exist for (5) with different *D*(*U*), see Fig. 10a in Sect. 4.3. The lower bound $c^*$ in Theorem 1 is often called the *minimum wave speed* as it represents the monotone nonnegative travelling wave solutions with the lowest wave speed (Murray 2002). Numerical simulations show that (2) with (3) and (5) indeed support smooth travelling wave solutions even though the nonlinear diffusivity function goes negative. Moreover, the speed relates to the initial condition, and the wave speed converges to the minimum wave speed $c^*$ as the initial condition limits to the Heaviside initial condition, see Fig. 2. We will also show the connection between the existence of smooth monotone nonnegative travelling wave solutions, the spectrum of a desingularised linearised operator associated with the travelling wave solutions, and the minimum wave speed $c^*$.

This manuscript is organised as follows. We prove Theorem 1 in Sect. 2 by using desingularisation techniques (Aronson 1980) and detailed phase plane analysis which have not been applied to (2) before. In Sect. 3, we determine the spectral properties of a desingularised linearised operator associated with the travelling wave solutions and show how the minimum wave speed $c^*$ is related to absolute instabilities (Sandstede 2002; Kapitula and Promislow 2013; Sherratt et al. 2014). Some interesting results for different nonlinear diffusivity functions with the same kinetic term (5) are discussed in Sect. 4. Here, we also discuss the implications of the analytical results for the discrete model. Note that throughout the manuscript all theoretical results are supported by high-quality numerical simulations of the continuum PDE model.

### Remark 1

Many essential mathematical questions related to, for instance, well-posedness, remain open for PDEs with forward–backward diffusion, i.e. models like (2) with nonlinear diffusivity functions that change sign. For instance, the well-studied Perona–Malik model (Perona and Malik 1990) from image analysis with forward–backward diffusion, but without a kinetic term, is ill-posed (Weickert 1988). See also Höllig (1983).

The ill-posedness of these PDEs with forward–backward diffusion can often be addressed by adding a small regularisation term, like a viscous regularisation term (Novick-Cohen and Pego 1991) or a nonlocal Cahn–Hilliard-type regularisation term (Pego and Penrose 1989). For the Perona–Malik model this was done, with another type of regularisation term, by Barenblatt et al. (1993). Interestingly, different regularisations can have different singular limits, in particular, when shock solutions are formed (see also Sect. 4.3). This is particularly interesting when you realise that most numerical schemes introduce some artificial regularisation. In other words, different numerical schemes can correctly yield different solutions (Witelski 1995). Also, recall that in the derivation of the continuum limit higher order terms were ignored. These higher order terms potentially have a regularising effect and can shed light on the “right” type of regularisation.

Since we are constructing smooth solutions in this manuscript, we do not address the question of well-posedness of (2).

## Existence of travelling wave solutions

### Transformation and desingularisation

We use a dynamical systems approach to analyse the second-order ODE (10) whose solutions that asymptote to ${\lim \nolimits _{z\rightarrow -\infty }}u=1$ and ${\lim \nolimits _{z\rightarrow \infty }}u=0$ correspond to travelling wave solutions of (2). Upon introducing $p:=D(u)du/dz$, (10) can be written as a singular system of first-order ODEs

12 $$\begin{aligned} \left\{ \begin{aligned} D(u)\frac{du}{dz}&=p,\\ D(u)\frac{dp}{dz}&=-cp-D(u)R(u). \end{aligned}\right. \end{aligned}$$

Travelling wave solutions of (2) now correspond to heteroclinic orbits of (12) connecting (1, 0) to (0, 0). Note that $p>0$ if $du/dz<0$ and $D(u)<0$. Thus, while we expect that the derivative of a travelling wave solution is always negative, *p* is not necessarily always negative.The nullclines of system (12) are given by $p=0$ and $-cp-D(u)R(u)=0$ with the constraint that $D(u)\ne 0$. However, *D*(*u*) vanishes when $u=\alpha$ and $u=\beta$ (7), and system (12) is thus undefined, or singular, along the lines $u=\alpha$ and $u=\beta$ (Simpson and Landman 2007). These lines are sometimes called *walls of singularities* (Pettet et al. 2000; Wechselberger and Pettet 2010; Harley et al. 2014a). Trajectories can potentially still cross through these walls at special points, sometimes referred to as *holes in the wall* (Pettet et al. 2000; Wechselberger and Pettet 2010; Harley et al. 2014a), when, in addition to $D(u)=0$, the right hand sides of the singular system also vanish (and if the holes in the wall are of the correct type (Wechselberger 2005; Wechselberger and Pettet 2010; Harley et al. 2014a)). These holes in the wall, and the trajectories crossing them, can often be linked to *folded singularities* and *canard solutions* upon embedding the singular system into higher-dimensional singularly perturbed systems with *folded critical manifolds*, we refer to Szmolyan and Wechselberger (2001), Wechselberger (2005), Wechselberger and Pettet (2010) and Harley et al. (2014a), and references therein, for more details on this now well-established theory. For system (12) the holes in the wall are $(\alpha ,0)$ and $(\beta ,0)$. To remove the singularities, we desingularise system (12) by introducing a stretched variable $\xi$ satisfying $D(u)d\xi =dz$ (Aronson 1980; Murray 2002; Sánchez-Garduño and Maini 1994; Harley et al. 2014a). Subsequently, system (12) becomes

13 $$\begin{aligned} \left\{ \begin{aligned}&\frac{du}{d\xi }=p,\\&\frac{dp}{d\xi }=-cp-D(u)R(u). \end{aligned}\right. \end{aligned}$$

**Fig. 3:**
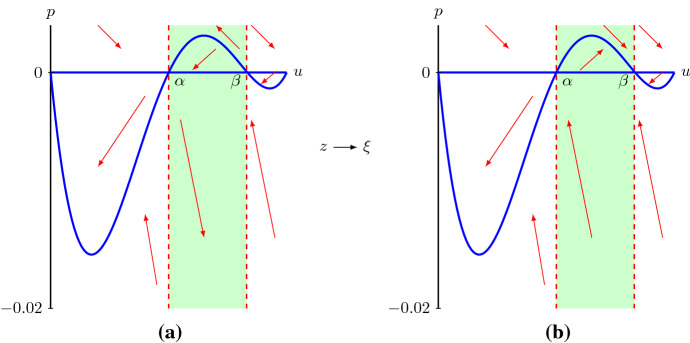
**a** The phase plane of system (12) with parameters $D_i=0.25$, $D_g=0.05$, $\lambda =0.75$ and $c=0.866$. The vertical dashed lines are the walls of singularities $u=\alpha$ and $u=\beta$ and the solid blue lines are nullclines. Red arrows show the orientation of the trajectories. **b** The phase plane of system (13) for the same parameter values and red lines are nullclines. For *u* in between $\alpha$ and $\beta$, the orientation of the trajectories is opposite compared to (**a**), while the orientation is the same for $u<\alpha$ and $u>\beta$ (colour figure online)

Here we see that the desingularisation changes the independent variable *z* in a nonlinear fashion, but it does not change the dependent variables (*u*, *p*). Consequently, the (*u*, *p*) phase planes of (12) and (13) will have the same trajectories but the “time” it takes to evolve along such a trajectory is different. In particular, when $D(u)>0$, $d\xi /dz>0$ and therefore trajectories on the phase planes of (12) and (13) have the same orientation. In contrast, when $D(u)<0$, $d\xi /dz<0$ and trajectories on the two phase planes are in the opposite direction, see Fig. 3. Therefore, heteroclinic orbits of (12) connecting (1, 0) to (0, 0) crossing the holes in the walls $(\alpha ,0)$ and $(\beta ,0)$, if they exist, are transformed and separated as heteroclinic orbits connecting (1, 0) to $(\beta ,0)$, $(\alpha ,0)$ to $(\beta ,0)$ and $(\alpha ,0)$ to (0, 0) of (13) and *vice versa*. Next, we will prove the existence of these heteroclinic orbits in system (13) for a range of wave speeds *c*, and then combine these heteroclinic orbits in system (13) as one global heteroclinic orbit in system (12).

### Phase plane analysis of the desingularised system

We first study the desingularised system (13). It has nullclines $p=0$ and

14 $$\begin{aligned} p=-\frac{D(u)R(u)}{c}. \end{aligned}$$

The intersections of the two nullclines give four equilibrium points: $(0,0),(1,0),(\alpha ,0),(\beta ,0)$.

#### Lemma 1

The equilibrium points (1, 0) and $(\alpha ,0)$ are saddles. The equilibrium point (0, 0) is a stable node if

15 $$\begin{aligned} c\ge 2\sqrt{D(0)R'(0)}=2\sqrt{\lambda D_i}=c^*, \end{aligned}$$

and a stable spiral otherwise. The equilibrium point $(\beta ,0)$ is a stable node if

16 $$\begin{aligned} c\ge 2\sqrt{D'(\beta )R(\beta )}, \end{aligned}$$

and a stable spiral otherwise.

#### Proof

The Jacobian of system (13) is

17 $$\begin{aligned} J(u,p)= & {} \left( \begin{matrix} 0 &{} 1 \\ -F(u) &{} -c \end{matrix}\right) ,\quad \text {where}\nonumber \\ F(u):= & {} \frac{d}{du}\left( D(u)R(u)\right) =D'(u)R(u)+D(u)R'(u), \end{aligned}$$

with *D*(*u*)*R*(*u*) the pointwise product of *D*(*u*) and *R*(*u*) and where we, as usual, omit the dot. The Jacobian has eigenvalues and eigenvectors

$$\begin{aligned} \lambda _{\pm }=\frac{-c\pm \sqrt{c^2-4F(u)}}{2},\quad E_{\pm }=(1,\lambda {\pm }). \end{aligned}$$

For the equilibrium point (1, 0) this reduces to

18 $$\begin{aligned} \lambda _{1\pm }=\frac{-c\pm \sqrt{c^2-4D(1)R'(1)}}{2},\quad E_{1\pm }=(1,\lambda _{1\pm }). \end{aligned}$$

The eigenvalues $\lambda _{1\pm }$ are real and of opposite sign since $D(1)=D_g>0$ and $R'(1)=-\lambda <0$. Thus (1, 0) is a saddle.

Similarly, the Jacobian of the equilibrium point $(\alpha ,0)$ has eigenvalues and eigenvectors

19 $$\begin{aligned} \lambda _{\alpha \pm }=\frac{-c\pm \sqrt{c^2-4D'(\alpha )R(\alpha )}}{2},\quad E_{\alpha \pm }=(1,\lambda _{\alpha \pm }). \end{aligned}$$

Knowing that $D'(\alpha )<0$ and $R(\alpha )>0$, $\lambda _{\alpha +}$ is real and positive and $\lambda _{\alpha -}$ is real and negative. Thus $(\alpha ,0)$ is a saddle.

The Jacobian of the equilibrium point (0, 0) has eigenvalues and eigenvectors

20 $$\begin{aligned} \lambda _{0\pm }=\frac{-c\pm \sqrt{c^2-4D(0)R'(0)}}{2},\quad E_{0\pm }=(1,\lambda _{0\pm }). \end{aligned}$$

The eigenvalues $\lambda _{0\pm }$ are real and negative if (15) holds since $D(0)=D_i>0$ and $R'(0)=\lambda >0$. Thus the equilibrium point (0, 0) is a stable node if (15) holds. Otherwise, $\lambda _{0\pm }$ are complex-valued with negative real parts and (1, 0) is a stable spiral.

Similarly, the Jacobian of equilibrium point $(\beta ,0)$ has eigenvalues and eigenvectors

21 $$\begin{aligned} \lambda _{\beta \pm }=\frac{-c\pm \sqrt{c^2-4D'(\beta )R(\beta )}}{2},\quad E_{\beta \pm }=(1,\lambda _{\beta \pm }). \end{aligned}$$

The eigenvalues $\lambda _{\beta \pm }$ are real and negative if (16) holds since $D'(\beta )>0$ and $R(\beta )>0$. Thus the equilibrium point $(\beta ,0)$ is a stable node if (16) holds. Otherwise, $\lambda _{\beta \pm }$ are complex-valued with negative real parts and $(\beta ,0)$ is a stable spiral. $\square$

#### Lemma 2

For $D_i>4D_g$, the thresholds of conditions (15) and (16) are ordered as

22 $$\begin{aligned} c^* > 2\sqrt{D'(\beta )R(\beta )}. \end{aligned}$$

#### Proof

The right hand side of (22) is given by

$$\begin{aligned} 2\sqrt{D'(\beta )R(\beta )}=2\sqrt{3\lambda (D_i-D_g)\beta (1-\beta )(\beta -\alpha )}. \end{aligned}$$

Since $c^*=2\sqrt{\lambda D_i}$, proving relation (22) is equivalent to proving

$$\begin{aligned} D_i > 3(D_i-D_g)\beta (1-\beta )(\beta -\alpha ), \end{aligned}$$

which is equivalent to proving

23 $$\begin{aligned} \frac{D_i}{D_i-D_g}> 3\beta (1-\beta )(\beta -\alpha ). \end{aligned}$$

Knowing that $2/3<\beta <1$ and $0<\beta -\alpha <2/3$ gives $3\beta (1-\beta )(\beta -\alpha )<2/3$. Since $D_i>4D_g$, we have that ${D_i}/{(D_i-D_g)}>1$ since $D_i>D_i-D_g$. Hence, (23) holds and thus (22) holds. $\square$

**Fig. 4:**
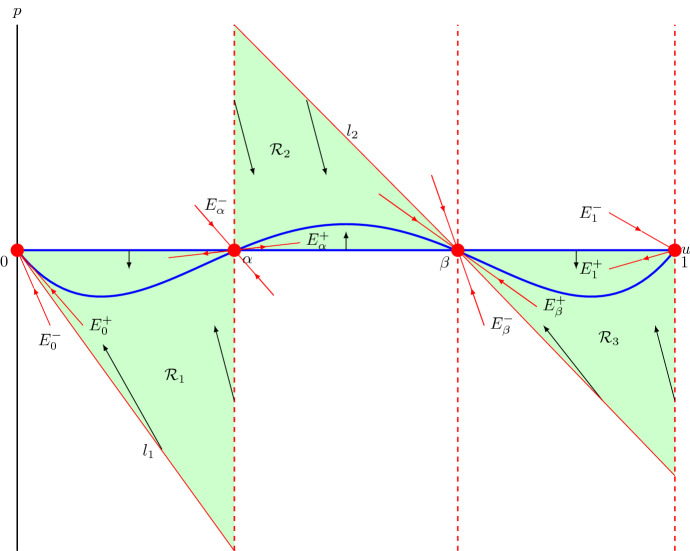
A qualitative phase plane of system (13). The three dashed lines are $u=\alpha$, $u=\beta$ and $u=1$. The blue lines are the nullclines $p=0$ and $p=-D(u)R(u)/c$. Region $\mathcal {R}_1$ is bounded by $p=0$, $u=\alpha$ and a straight line $l_1$ with negative slope passing through (0, 0). Region $\mathcal {R}_2$ is bounded by $p=0$, $u=\alpha$ and a straight line $l_2$ with negative slope passing through $(\beta ,0)$. Region $\mathcal {R}_3$ is bounded by $p=0$, $u=1$ and $l_2$ (colour figure online)

For $c<c^*$, (0, 0) becomes a spiral node and hence we expect trajectories approaching (0, 0) to become negative which in the end would lead to travelling wave solutions becoming negative. Therefore, we now assume that $c\ge c^*$. To prove the existence of heteroclinic orbits between the equilibrium points, we construct invariant regions in the phase plane from which trajectories cannot leave, so that the Poincaré–Bendixson theorem can be applied (Jordan and Smith 1999), see Fig. 4. The slope of nullcline (14) is $\chi (u)=-F(u)/c$, where *F*(*u*) is given by (17), while the slope of the unstable eigenvector of (1, 0) is $\lambda _{1+}$, see (18). We thus have

24 $$\begin{aligned} \lambda _{1+}-\chi (1)= & {} \frac{-c+\sqrt{c^2-4D(1)R'(1)}}{2}+\frac{1}{c}D(1)R'(1)\nonumber \\= & {} \frac{c\sqrt{c^2-4D(1)R'(1)}-\left( c^2-2D(1)R'(1)\right) }{2c}\nonumber \\= & {} \frac{\sqrt{c^4-4c^2D(1)R'(1)}-\sqrt{c^4-4c^2D(1)R'(1)+4\left( D(1)R'(1)\right) ^2}}{2c}<0.\nonumber \\ \end{aligned}$$

That is, the unstable eigenvector of (1, 0) has a smaller slope than nullcline (14) at (1, 0). In other words, the trajectory leaving (1, 0) with decreasing *u* initially lies above the nullcline (14).

Similarly, the slope of the unstable eigenvector of $(\alpha ,0)$ is $\lambda _{\alpha +}$, see (19). We have, after similar computation as (24), $\lambda _{\alpha +}-\chi (\alpha )<0$. Thus, the unstable eigenvector of $(\alpha ,0)$ has a smaller slope than nullcline (14) at $(\alpha ,0)$. Therefore, the trajectory leaving $(\alpha ,0)$ with decreasing *u* initially lies above the nullcline (14), while the trajectory leaving $(\alpha ,0)$ with increasing *u* initially lies below the nullcline (14).

Under condition (15), the least negative slope of the stable eigenvectors of equilibrium point (0, 0) is $\lambda _{0+}$, see (20). This gives, after a similar computation as (24), $\lambda _{0+}-\chi (0)<0$. Thus, both eigenvectors of (0, 0) have slopes that are more negative than nullcline (14) at (0, 0). In other words, the eigenvectors of (0, 0) initially lie under the nullcline (14) for $u>0$.

Similarly, under condition (16), the least negative slope of the stable eigenvectors of $(\beta ,0)$ is $\lambda _{\beta +}$, see (21). This gives $\lambda _{\beta +}-\chi (\beta )<0$. Thus, both eigenvectors have slopes that are more negative than nullcline (14) at $(\beta ,0)$. Therefore, the trajectory moving in $(\beta ,0)$ with decreasing *u* initially lies under the nullcline (14) for $u>\beta$, while they lie above the nullcline (14) for $u<\beta$, see also Fig. 4.

Next, we consider the region $\mathcal {R}_1$ bounded by $p=0$, $u=\alpha$ and a straight line $l_1$ through (0, 0) with a negative slope $\mu _1$. We aim to prove that for $c\ge c^*$, there always exists a slope $\mu _1$ so that no trajectories in region $\mathcal {R}_1$ can cross through its boundaries. Trajectories starting on $p=0$ have negative vertical directions since $du/d\xi =p=0$ and $dp/d\xi =-D(u)R(u)<0$ for $u\in (0,\alpha )$. Thus, trajectories in $\mathcal {R}_1$ cannot cross through $p=0$. Trajectories starting on $u=\alpha$ with negative *p* values point into region $\mathcal {R}_1$ since $du/d\xi =p<0$ and $dp/d\xi =-cp>0$. Trajectories starting on $l_1$ satisfy $p=\mu _1 u$, and they point into $\mathcal {R}_1$ only if

$$\begin{aligned} \frac{dp}{du}\Bigr |_{p=\mu _1u}=-c-\frac{D(u)R(u)}{\mu _1u}\le \mu _1,\quad \text {for}\quad u\in (0,\alpha ). \end{aligned}$$

After rearranging and recalling that $\mu _1<0$, we obtain

25 $$\begin{aligned} \mu _1(\mu _1+c)\le -\frac{D(u)R(u)}{u} = -\lambda D(u)(1-u),\quad \text {for}\quad u\in (0,\alpha ). \end{aligned}$$

#### Lemma 3

For $c\ge c^*$, there exists a $\mu _1$ such that inequality (25) is valid for any $u\in (0,\alpha )$.

#### Proof

Proving inequality (25) is equivalent to proving

26 $$\begin{aligned} \mu _1(\mu _1+c)\le - \lambda \sup _{u\in (0,\alpha )} D(u)(1-u). \end{aligned}$$

The left hand side of inequality (26) is minimal when $\mu _1=-c/2$. Setting $\mu _1=-c/2$ and substituting into inequality (26) gives a lower bound

27 $$\begin{aligned} c_1=2 \sqrt{\lambda } \sup _{u\in (0,\alpha ]}\sqrt{D(u)(1-u)}, \end{aligned}$$

such that (26) holds for $c\ge c_1$. The right hand side of (27) gives

$$\begin{aligned} 2 \sqrt{\lambda } \sup _{u\in (0,\alpha )}\sqrt{D(u)(1-u)}=2\sqrt{\lambda D(0)}=2\sqrt{\lambda D_i}, \end{aligned}$$

since *D*(*u*) and $(1-u)$ are both decreasing functions on $u\in (0,\alpha )$. Thus, $c_1=c^*$. Hence, for $c\ge c^*$, inequality (26) is valid for $\mu _1=-c/2$. $\square$

Knowing that for $c\ge c^*$ inequality (25) is valid, trajectories on $l_1$ with $\mu _1=-c/2$ point into region $\mathcal {R}_1$. Thus, based on the Poincaré-Bendixson theorem (Jordan and Smith 1999), the observation that the derivative of *u* is negative in the region $\mathcal {R}_1$ (preventing the existence of a homoclinic orbit) and the absence of fixed points in the interior of $\mathcal {R}_1$ (preventing the existence of a limit cycle), the trajectory leaving from the equilibrium point $(\alpha ,0)$ with decreasing *u* and decreasing *p* must connect with the equilibrium point (0, 0) without going negative in *u*.

Similarly, we consider the region $\mathcal {R}_2$ bounded by $p=0$, $u=\alpha$ and a straight line $l_2$ through $(\beta ,0)$ with a negative slope $\mu _2$, and the region $\mathcal {R}_3$ bounded by $p=0$, $u=1$ and $l_2$. Trajectories starting on $p=0$ have positive vertical directions for $u\in (\alpha , \beta )$ since $du/d\xi =p=0$ and $dp/d\xi =-D(u)R(u)>0$ and they have negative vertical directions since for $u\in (\beta ,1)$, $du/d\xi =0$ and $dp/d\xi =-D(u)R(u)<0$. Trajectories starting on $u=\alpha$ with positive *p* point into region $\mathcal {R}_2$ since $du/d\xi =p>0$ and $dp/d\xi =-cp<0$. Similarly, trajectories starting on $u=1$ with negative *p* point into region $\mathcal {R}_3$. In addition, requiring the existence of a slope $\mu _2$ such that trajectories starting on $l_2$ point into regions $\mathcal {R}_2$ and $\mathcal {R}_3$ leads to the condition

28 $$\begin{aligned} \mu _2(\mu _2+c)\le -\frac{D(u)R(u)}{u-\beta }=-3(D_i-D_g)(u-\alpha )R(u), \quad \text {for} \quad u\in (\alpha ,1). \end{aligned}$$

#### Lemma 4

For $c\ge c^*$, there exists a $\mu _2$ such that inequality (28) is valid for any $u\in (\alpha ,1)$.

#### Proof

The proof of Lemma 4 is analogous to the proof of Lemma 3 and we will omit some of the details. Again, there exists a lower bound

$$\begin{aligned} c_2 =2 \sqrt{3(D_i-D_g)} \sup _{u\in (\alpha ,1)}\sqrt{(u-\alpha )R(u)}, \end{aligned}$$

such that (28) holds for $c\ge c_2$. Next, we show that $c_2<c^*$. That is, we show that

$$\begin{aligned} 2\sqrt{\lambda D_i}> 2 \sqrt{3(D_i-D_g)} \sup _{u\in (\alpha ,1)}\sqrt{(u-\alpha )R(u)}. \end{aligned}$$

This is equivalent to proving $D_i/(D_i-D_g)>3u(1-u)(u-\alpha )$ for $u\in (\alpha ,1)$. Noticing that $u-\alpha <2/3$, and $u(1-u)\le 1/4$, we obtain $3u(1-u)(u-\alpha )<1/2$. Subsequently, we have

$$\begin{aligned} \frac{D_i}{D_i-D_g}>1>\frac{1}{2}>3u(1-u)(u-\alpha ), \end{aligned}$$

since $D_i>4D_g$ by assumption. Thus, $c_2<c^*$. $\square$

Knowing that for $c\ge c^*$ the inequality (28) is valid, trajectories on $l_2$ in between $\alpha$ and $\beta$ point into region $\mathcal {R}_2$. Thus, based on the Poincaré–Bendixson theorem (Jordan and Smith 1999), the trajectory leaving from the equilibrium point $(\alpha ,0)$ with increasing *u* and increasing *p* must connect with the equilibrium point $(\beta ,0)$. Analogously, the trajectory leaving from the equilibrium point (1, 0) with decreasing *u* and decreasing *p* must connect with the equilibrium point $(\beta ,0)$.

In summary, for $c\ge c^*$ there exist heteroclinic orbits connecting (1, 0) to $(\beta ,0)$, $(\alpha ,0)$ to $(\beta ,0)$ and $(\alpha ,0)$ to (0, 0) in system (13). Since trajectories in $u\in (0,\alpha )\cup (\beta ,0)$ in system (12) are the same, and have the same orientation, as in system (13), there exist trajectories connecting (1, 0) to the hole in the wall $(\beta ,0)$ and trajectories connecting the hole in the wall $(\alpha ,0)$ to (0, 0) in system (12). For $u\in (\alpha ,\beta )$, trajectories of system (12) move in the opposite direction compared to (13), see Fig. 3. The trajectory leaving from $(\alpha ,0)$ with increasing *u*, positive *p* and connecting to $(\beta ,0)$ in system (13) becomes a trajectory leaving from $(\beta ,0)$ with decreasing *u*, positive *p* and connecting to $(\alpha ,0)$ in system (12). Thus, there exists an orbit connecting $(\beta ,0)$ to $(\alpha ,0)$ in system (12). Combining the above, we get that for $c \ge c^*$, there exists a heteroclinic orbit with $u\ge 0$ connecting (1, 0) to (0, 0) passing through holes in the walls $(\alpha ,0)$ and $(\beta ,0)$ in system (12), however, see Remark 2. Hence, there exist smooth monotone travelling wave solutions of (2) with positive speed $c\ge c^*$. This completes the proof of Theorem 1.

**Fig. 5:**
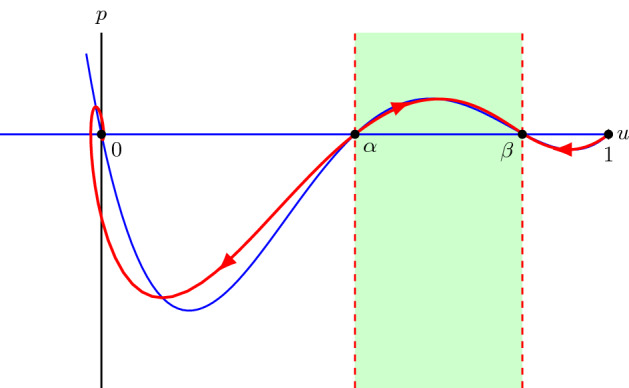
Phase plane of system (13) with parameters $D_i=0.25$, $D_g=0.05$, $\lambda =0.75$ and $c=0.4$. The latter is smaller than $c^*\approx 0.866$ but larger than $2\sqrt{D'(\beta )R(\beta )} \approx 0.289$. The blue lines are the nullclines $p=0$ and $p=-D(u)R(u)/c$. The red lines are the heteroclinic orbits connecting (0, 0), $(\alpha ,0)$, $(\beta ,0)$, and (1, 0) (colour figure online)

For $2\sqrt{D'(\beta )R(\beta )}< c<c^*$ the equilibrium point $(\beta ,0)$ of the desingularised system (13) is still a stable node, while (0, 0) is a stable spiral, see Lemma 1. We can use similar techniques as above to show that system (13) still possesses heteroclinic orbits connecting (1, 0) to $(\beta ,0)$, $(\alpha ,0)$ to $(\beta ,0)$ and $(\alpha ,0)$ to (0, 0), see also Fig. 5. However, this latter heteroclinic orbit now spirals into (0, 0). Consequently, also for $2\sqrt{D'(\beta )R(\beta )}< c<c^*$ there exists a heteroclinic orbit connecting (1, 0) to (0, 0) passing through holes in the walls $(\alpha ,0)$ and $(\beta ,0)$ in system (12). However, these correspond to smooth travelling wave solutions of (2) with (3) and (5) that are not monotone and instead oscillate around 0. These solutions are not biologically relevant as *U* represents the population density in the discrete model and thus cannot be negative.

For $0<c<2\sqrt{D'(\beta )R(\beta )}$, $(\beta ,0)$ becomes a stable spiral in (13) and hence trajectories in system (12) can no longer pass through this hole in the wall, i.e. the hole in the wall is not of the correct type (Harley et al. 2014a). That is, (2) with (3) and (5) do not support smooth travelling wave solutions for $0<c<2\sqrt{D'(\beta )R(\beta )}$. Note that there may exist shock-fronted travelling wave solutions, however, we are not interested in such solutions in this manuscript as (0, 0) is still a stable spiral of (13) and thus again yields solutions that are not biologically relevant. See Sect. 4.3 for a further discussion related to shock-fronted travelling wave solutions supported by (2).

#### Remark 2

It is important to note that combining the three heteroclinic orbits in the desingularised system (13) to get the global orbit in the original system (12) is not trivial. Although the relationship between the trajectories, and their orientation, in the two systems is clear, we still need to prove that orbits are able to pass through the holes in the wall in (12) by, for instance, using canard theory (Szmolyan and Wechselberger 2001; Wechselberger 2005, 2012). Roughly speaking, we embed the original ODE (10) into a larger class of problems by adding a higer order perturbation term with a small parameter $0\le \epsilon \ll 1$. Subsequently, rather than obtaining the two-dimensional system (12), we have a higher-dimensional system which has a *slow–fast* structure that can be studied by geometric singular perturbation theory (Jones 1995). Most notably, the two-dimensional system (12) would become the reduced problem of the higher-dimensional system in the singular limit $\epsilon \rightarrow 0$ and it is constraint on a folded critical manifold. With canard theory we can show the existence of solutions crossing through the holes in the wall (or folded canard points) in the higher-dimensional system for $0\le \epsilon \ll 1$. As this is by now relatively standard and straightforward, we decide to omit the details and instead refer to Szmolyan and Wechselberger (2001), Wechselberger (2005) and Wechselberger (2012), and references therein.

## Stability analysis

We showed that, similar to the Fisher–KPP equation (Harley et al. 2015, e.g.), (2) with (3) and (5) supports smooth travelling wave solutions for $c > 2\sqrt{D'(\beta )R(\beta )}$, but that only the travelling wave solutions with $c \ge c^*$ (11) have nonnegative densities. The minimal wave speed for the Fisher–KPP equation is closely related to the onset of absolute instabilities.^1^ Roughly speaking, absolute instabilities imply that perturbations to a travelling wave solution (in an appropriate Sobolev space that will be discussed further on) will grow for all time and at every point in space (Sherratt et al. 2014). These instabilities are related to the absolute spectrum of the linear operator associated with the travelling wave solution and is fully determined by the asymptotic behaviour ($z \rightarrow \pm \infty$) of the travelling wave solution (Kapitula and Promislow 2013; Sandstede 2002). Note that the absolute spectrum is, strictly speaking, not part of the spectrum of the linear operator. However, it gives an indication on how far the essential spectrum can be shifted to the left upon using a weighted Sobolev space (Kapitula and Promislow 2013; Sandstede 2002). Consequently, if parts of the absolute spectrum lie in the right half plane, then the essential spectrum cannot be fully weighted into the open left half plane, and the associate solution is hence absolutely unstable.^2^ The travelling wave solutions of (2) with (3) and (5) as constructed in Sect. 2 asymptote to 0 and 1 and the nonlinear diffusivity function *D*(*U*) is positive near $U=0$ and $U=1$, see (6). That is, near these points (2) with (3) and (5) has a Fisher–KPP imprint and we therefore expect that the minimal wave speed $c^*$ of (2) is also closely related to the onset of absolute instabilities. In other words, we expect that the travelling wave solutions of (2) with (3) and (5) are absolutely unstable for $2\sqrt{D'(\beta )R(\beta )}< c<c^*$. Therefore, we expect perturbations to these travelling wave solutions to always grow and we will never observe travelling waves with these speeds in, for instance, numerical simulations. Consequently, while (2) with (3) and (5) support these biologically irrelevant travelling wave solutions that go negative, they will never be observed and thus do not effect the feasibility of the model.

Starting with a travelling wave solution ${\hat{u}}(z)$, we add a small perturbation *q*(*z*, *t*) and substitute $u(z,t)={\hat{u}}(z)+q(z,t)$ into (9) and, upon ignoring higher-order perturbative terms $\mathcal {O}(q^2)$, we get

29 $$\begin{aligned} \begin{aligned} \frac{\partial q}{\partial t}={\mathcal {L}} q\,, \qquad \text {with} \quad \mathcal {L}q := \frac{\partial }{\partial z}\left( \frac{\partial }{\partial z}\left( D({\hat{u}})q \right) \right) + c \frac{\partial q}{\partial z} + \left( R'({\hat{u}})\right) q\,. \end{aligned} \end{aligned}$$

The associated eigenvalue problem, which is obtained by setting $q(z,t)=e^{\varLambda t}q(z)$, is given by

30 $$\begin{aligned} \mathcal {L} q=\varLambda q. \end{aligned}$$

Upon introducing $s:= \dfrac{d}{dz} \left( D({\hat{u}}) q \right)$, the eigenvalue problem (30) can be written as a system of first order singular ODEs

31 $$\begin{aligned} \mathcal {T}(\varLambda )\left( \begin{aligned}&q\\&s \end{aligned}\right)&:=\left( D({\hat{u}})\dfrac{d}{dz}-A(z;\varLambda )\right) \left( \begin{aligned}&q\\&s \end{aligned}\right) = 0\,, \,\, \mathrm{where} \,\, \\ \nonumber A(z;\varLambda )&:=\begin{pmatrix} -\mathcal {B}(z)&{}1\\ c \mathcal {B}(z) + D({\hat{u}})\left( \varLambda - R'({\hat{u}})\right) &{}-c \end{pmatrix}, \end{aligned}$$

with $\mathcal {B}=D'({\hat{u}})\dfrac{d{\hat{u}}}{dz}$. We desingularise the above system by making (essentially) the same transformation that we made to get to equation (13). That is, we define $\xi$ so that $D({\hat{u}})d\xi = dz$ and (31) becomes

32 $$\begin{aligned} \tilde{\mathcal {T}}(\varLambda )\left( \begin{aligned}&q\\&s \end{aligned}\right) :=\left( \frac{d}{d\xi }-A(\xi ;\varLambda )\right) \left( \begin{aligned}&q\\&s \end{aligned}\right) = 0\,, \end{aligned}$$

with *A* and $\mathcal {B}$ as above, but with the observation that $d {\hat{u}}/dz = (d{\hat{u}}/d\xi )/D({\hat{u}})$. We have shown in the previous section that $d {\hat{u}}/dz$ is a smooth bounded function, and, as such, (32) is a perfectly well-defined system of equations on $\mathbb {R}$. In particular, it is well-posed and the usual analysis for continuous and absolute spectrum will apply here (though the introduction of the variable $\xi$ means that for certain parts of the linear system the flow will go in the opposite direction—but this will not happen in the far field $z \rightarrow \pm \infty$).

We call the operator $\tilde{\mathcal {T}}$ spectrally stable if the spectrum is in the open left half plane and unstable otherwise, with the possible exception of 0. The spectrum of $\tilde{\mathcal {T}}$ naturally breaks up into two sets, the point spectrum and the essential spectrum (Kapitula and Promislow 2013; Sandstede 2002). Roughly speaking, the essential spectrum of the operator deals with the behaviour in the far field, while the point spectrum contains information about more localised solutions to the eigenvalue problem.

Obviously the spectral properties $\tilde{\mathcal {T}}$ depend on the domain we choose for it. A natural choice is the space of square integrable functions whose first (weak) derivative (in *z*) is also square integrable, that is, the Sobolev space $\mathbb {H}^1(\mathbb {R})$. Another choice is the related one-sided weighted space $\mathbb {H}^1_\nu (\mathbb {R})$ defined as $q \in \mathbb {H}^1_\nu (\mathbb {R})$ if and only if $e^{\nu z} q \in \mathbb {H}^1(\mathbb {R})$ (Kapitula and Promislow 2013; Sattinger 1977). For positive $\nu$ the weight forces *q* to decay at a rate faster than $e^{-\nu z}$ as $z\rightarrow \infty$ while it is allowed to grow exponentially, but at a rate less than $e^{-\nu z}$ as $z \rightarrow -\infty$. That is, the weight provides information whether solutions to (32) are more prone to growing at plus or minus infinity (Davis et al. 2017). The weighting of $\mathbb {H}^1(\mathbb {R})$ shifts the essential spectrum (Kapitula and Promislow 2013), so an operator can be spectrally unstable with respect to perturbations in $\mathbb {H}^1(\mathbb {R})$, while it is stable with respect to perturbations in an appropriately weighted space $\mathbb {H}^1_\nu (\mathbb {R})$. This is, for instance, the case for the linearised Fisher–KPP equation and the linearisation of a particular Keller–Segel model (Davis et al. 2017, 2019). The absolute spectrum of an operator is not affected by the weighting of the space and gives an indication on how far the essential spectrum can be weighted (as the absolute spectrum is always to the left of the rightmost boundary of the essential spectrum (Davis et al. 2017)). In other words, if the absolute spectrum of a solution contains part of the right half plane then the essential spectrum cannot be weighted into the open left half plane and the solution is said to be absolutely unstable.

The unweighted essential spectrum and the absolute spectrum of the operator $\tilde{\mathcal {T}}$ are determined by its asymptotic behaviour, since the operator is a relatively compact perturbation of the limiting operator as $z = \pm \infty$ (Kapitula and Promislow 2013). Therefore, we define the asymptotic matrices

$$\begin{aligned} A_+(\varLambda ):=\lim _{z\rightarrow +\infty }A(z,\varLambda )= \begin{pmatrix} 0&{}1\\ D(0)(\varLambda -R'(0))&{}-c \end{pmatrix}, \end{aligned}$$

and

$$\begin{aligned} A_-(\varLambda ):=\lim _{z\rightarrow -\infty }A(z,\varLambda )= \begin{pmatrix} 0&{}1\\ D(1)(\varLambda -R'(1))&{}-c \end{pmatrix}. \end{aligned}$$

More specifically, for the problem at hand the boundary of the unweighted essential spectrum of $\tilde{\mathcal {T}}$ is determined by those $\varLambda$ for which $A_{\pm }(\varLambda )$ has a purely imaginary eigenvalue. In contrast, the absolute spectrum at $\pm \infty$ is determined by those $\varLambda$ for which the eigenvalues of $A_{\pm }(\varLambda )$ have the same real part (Sandstede 2002). The eigenvalues of $A_+$ are

33 $$\begin{aligned} \mu _{+}^{\pm }=\frac{-c\pm \sqrt{c^2+4D(0)(\varLambda -R'(0))}}{2}, \end{aligned}$$

and those of $A_{-}$ are

34 $$\begin{aligned} \mu _{-}^{\pm }=\frac{-c\pm \sqrt{c^2+4D(1)(\varLambda -R'(1))}}{2}. \end{aligned}$$

**Fig. 6:**
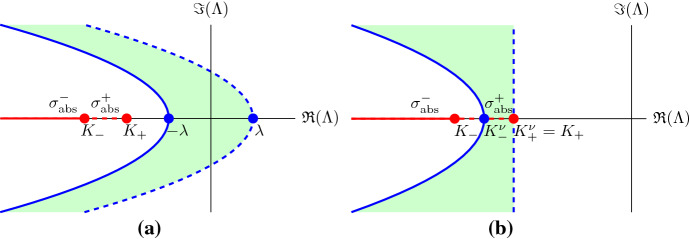
**a** The unweighted essential spectrum and the absolute spectrum of the linear operator $\tilde{\mathcal {T}}$ for $c>c^*$. The boundary of the unweighted essential spectrum is determined by the dispersion relations of $A_+$ (dashed blue curve) and $A_-$ (solid blue curve) and the green region is the interior of the unweighted essential spectrum. The solid red line is the absolute spectrum $\sigma ^+_{\text {abs}}$ (35), while the dashed red line is the absolute spectrum $\sigma ^+_{\text {abs}}$ (35). **b** The unweighted essential spectrum is, for a weight $\nu =c/(2D(0))$ with $c\ge c^*$, shifted to the rightmost boundary of the absolute spectrum $\sigma ^+_{\text {abs}}$ (colour figure online)

Hence, the boundary of the unweighted essential spectrum is given by the so-called dispersion relations

$$\begin{aligned} \varLambda _+=-D(0)k^2+ick+R'(0), \quad \mathrm{and} \quad \varLambda _-=-D(1)k^2+ick+R'(1), \end{aligned}$$

where $k\in \mathbb {R}$ and where $\mu _+^+=i D(0)k$ and $\mu _-^+=i D(1)k$ are the purely imaginary spatial eigenvalue of $A_\pm$. These dispersion relations form two parabolas, opening leftward and intersecting the real axis at $R'(0)=\lambda >0$ and $R'(1)=-\lambda <0$, see Fig. 6. That is, all travelling wave solutions of (2) with (3) and (5) have unweighted essential spectrum in the right half plane.

From (33) we get that the absolute spectrum at $+\infty$ is given by

35 $$\begin{aligned} \sigma _{\text {abs}}^+=\left\{ \varLambda \in \mathbb {R}\ \bigg |\ \varLambda <-\frac{c^2}{4D(0)}+R'(0) = -\frac{c^2}{4D_i}+\lambda =: K_+ \right\} . \end{aligned}$$

Similarly, from (34) we get that the absolute spectrum at $-\infty$ is given by

36 $$\begin{aligned} \sigma _{\text {abs}}^-=\left\{ \varLambda \in \mathbb {R}\ \bigg |\ \varLambda <-\frac{c^2}{4D(1)}+R'(1) = -\frac{c^2}{4D_g}-\lambda =: K_-\right\} . \end{aligned}$$

That is, $\sigma _{\text {abs}}^-$ is always fully contained in the open left half plane including the origin, while $\sigma _{\text {abs}}^+$ is only fully contained in the open left half plane including the origin for $c\ge c^*=2\sqrt{\lambda D_i}$, see Fig. 6.

The essential spectrum in the weighted space $\mathbb {H}^1_\nu (\mathbb {R})$ is determined by the operator

$$\begin{aligned} \mathcal {T}^\nu (\varLambda )\left( \begin{aligned}&q\\&s \end{aligned}\right) :=\left( D({\hat{u}})\frac{d}{dz}-\left( A(z;\varLambda )+ D({\hat{u}}) \nu I\right) \right) \left( \begin{aligned}&q\\&s \end{aligned}\right) = 0\,, \end{aligned}$$

or

$$\begin{aligned} \tilde{\mathcal {T}}^\nu (\varLambda )\left( \begin{aligned}&q\\&s \end{aligned}\right) :=\left( \frac{d}{d\xi }-\left( A(\xi ;\varLambda )+ D({\hat{u}}) \nu I\right) \right) \left( \begin{aligned}&q\\&s \end{aligned}\right) = 0\,, \end{aligned}$$

see Kapitula and Promislow (2013), and the weighted asymptotic matrices are

$$\begin{aligned} A_+^\nu (\varLambda )=A_+(\varLambda ) + D(0)\nu I= \begin{pmatrix} D(0) \nu &{}1\\ D(0)(\varLambda -R'(0))&{}-c+ D(0) \nu \end{pmatrix}, \end{aligned}$$

and

$$\begin{aligned} A_-^\nu (\varLambda )=A_-(\varLambda ) + D(1) \nu I= \begin{pmatrix} D(1) \nu &{}1\\ D(1)(\varLambda -R'(1))&{}-c+ D(1) \nu \end{pmatrix}. \end{aligned}$$

Hence, the boundary of the essential spectrum in the weighted space is given by the dispersion relations

$$\begin{aligned} \varLambda _+^\nu= & {} -D(0)k^2+i(c-2D(0)\nu )k+D(0)\nu ^2-c\nu +R'(0),\\ \varLambda _-^\nu= & {} -D(1)k^2+i(c-2D(1)\nu )k+D(1)\nu ^2-c\nu +R'(1). \end{aligned}$$

These dispersion relations still form two parabolas opening leftward and the intersections with the real axis now depend on $\nu$. We define the intersection of $\varLambda _+^\nu$ with the real axis as $K_+^{\nu }:=D(0)\nu ^2-c\nu +R'(0)$, and the intersection of $\varLambda _-$ on the real axis as $K_-^{\nu }:=D(1)\nu ^2-c\nu +R'(1)$. For $2\sqrt{D'(\beta )R(\beta )}<c<c^*$, $K_+^{\nu }$ is positive for all weights $\nu$, that is, $\varLambda _+^\nu$ always has a positive intersection on the real axis. In other words, for $2\sqrt{D'(\beta )R(\beta )}<c<c^*$ and in any weighted space $\mathbb {H}^1_\nu (\mathbb {R})$, parts of the boundary of the weighted essential spectrum lie in the open right half plane. For speed $c\ge c^*$, there exists a range of weights

37 $$\begin{aligned} \nu \in \left( \frac{c-\sqrt{c^2-(c^*)^2}}{2D(0)},\frac{c+\sqrt{c^2+(c^*)^2}}{2D(0)}\right) \end{aligned}$$

such that $K_+^\nu <0$, that is, $\varLambda _+$ has a negative intersection with the real axis. Furthermore, $K_-^\nu <K_+^\nu$. Therefore, for $c\ge c^*$, the unweighted essential spectrum is shifted into the open left half plane for weights in the above range (37). Furthermore, when $\nu =c/(2D(0))$, $K_+^\nu$ reaches its minimum, which coincides with $K_+$, the rightmost boundary of the absolute spectrum $\sigma ^+_{abs}$ (35). Note that $\nu =c/(2D(0))$ is the ideal one-sided weight (Davis et al. 2017), i.e. the weight that shifts the right most boundary of the essential spectrum furthest into the left half plane (since $\sigma ^+_{abs}$ is to the right of $\sigma ^-_{abs}$). See Fig. 6.

In conclusion, the operator $\tilde{\mathcal {T}}$ is absolutely unstable for $2\sqrt{D'(\beta )R(\beta )}<c<c^*$ and no weights exist to shift its unweighted essential spectrum into the open left half plane. In contrast, the absolute spectrum of $\tilde{\mathcal {T}}$ with speed $c\ge c^*$ is fully contained in the open left half plane including the origin and weights can be found that shift the unweighted essential spectrum into this region.

### Remark 3

While the desingularised operator $\tilde{\mathcal {T}}$ (32) is well-posed, the original eigenvalue operator $\mathcal {L}$ (30) has a forward–backward diffusion part and is therefore not. However, we do find the travelling wave solutions numerically in parameter regimes in accordance with the stable spectrum for (32). Lastly, we note that the travelling wave solution ${\hat{u}}$ consists of three heteroclinic orbits in the desingularised variable $\xi$, and while the asymptotic matrices related to the holes in the wall at $\alpha$ and $\beta$

$$\begin{aligned} \left. \begin{pmatrix} - D'({\hat{u}}) \dfrac{d {\hat{u}}}{d z} &{}1\\ c D'({\hat{u}}) \dfrac{d {\hat{u}}}{d z}&{}-c \end{pmatrix} \right| _{{\hat{u}}=\alpha , {\hat{u}} = \beta } \,. \end{aligned}$$

are not Fredholm since they have a zero eigenvalue, the corresponding constant solutions (i.e. $u = \alpha , \beta$) are not fixed points of the original travelling wave Eq. (10). So, these points are not really to be considered in the far field in terms of the variable *z*. It remains to be seen whether or not the asymptotic matrices in $\xi$ contribute to stability or instability of the travelling wave solutions ${\hat{u}}$ in *z*. Though, as we have mentioned above, numerical solutions to the travelling wave solutions have been found, so it appears as though, for some parameter regimes at least, they do not destabilise the wave.

## Summary and future work

### Summary of results

We started this manuscript with a lattice-based discrete model introduced in Johnston et al. (2017) that explicitly accounts for differences in individual and collective cell behaviour. Based on Johnston et al. (2017), the discrete model has the continuous description (2) obtained by using truncated Taylor series in the continuum limit. Our analysis focused on the case where $D_i>4D_g$ so that we can obtain a convex nonlinear diffusivity function *D*(*U*), given by (3), which changes sign twice in our domain of interest. Furthermore, the assumption of equal proliferation rates and zero death rates leads to a logistic kinetic term *R*(*U*), given by (5). The associated numerical simulations of (2) with (3) and (5), see Fig. 2, provided evidence of the existence of smooth monotone travelling wave solutions. To study these travelling wave solutions of (2), we used a travelling wave coordinate $z=x-ct$ and looked for stationary solutions in the moving frame. Consequently, (2) was transformed into the singular second-order ODE (10) which we transformed into a singular system of first-order ODEs (12). To remove the singularities, we used the stretched variable $D(u)d\xi =dz$ and transformed (12) into system (13). Next, we analysed the phase plane of the desingularised system (13) and proved the existence of heteroclinic orbits connecting the equilibrium points $(0,0),(\alpha ,0),(\beta ,0)$ and (1, 0) for wave speeds $c\ge c^*$, given by (11). Subsequently, based on the relation between the phase planes of (12) and (13), we proved the existence of a heteroclinic orbit in (12) connecting the equilibrium points (1, 0) and (0, 0) passing through $(\alpha ,0)$ and $(\beta ,0)$, that are special points on the phase plane called a hole in the wall of singularities. That is, we proved the existence of smooth monotone travelling wave solutions of (2) for $c\ge c^*$. In the end, we showed that the linear operator $\tilde{\mathcal {T}}$ (32), associated with the travelling wave solutions of (2), with wave speed $c<c^*$ is absolutely unstable, which in turn explained that the numerical simulations only provided travelling wave solutions with wave speeds $c\ge c^*$.

Based on our analysis, one-dimensional agent density profiles in the discrete model will eventually spread with a speed $c\ge c^*$ if the two types of agents have equal proliferation rates, zero death rates and different diffusivities satisfying $D_i>4D_g$. Notice that $c^*=2\sqrt{\lambda D_i}$, hence, the lowest speed for the travelling wave only relates to the diffusivity of individuals and is independent of the diffusivity of the grouped agents. That is, the diffusivity of grouped agents which is smaller than that of isolated agents ($D_i>4D_g$) does not give restrictions for the lowest speed of the moving front. Consequently, we infer that the speed of invasion processes for organisms, for instance, cells, is mainly determined by the behaviour of individuals. Furthermore, the Fisher–KPP equation also has a minimum wave speed for the existence of smooth monotone travelling wave solutions (Kolmogorov et al. 1937; Fife 2013). Hence, a discrete mechanism of invasion processes considering the differences in individual and collective behaviours can lead to a macroscopic behaviour similar to that observed in the discrete mechanism with no differences in isolated and grouped agents.

**Fig. 7:**
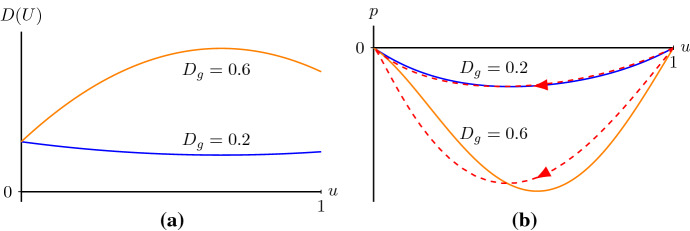
**a** *D*(*U*) with $D_i=0.25$ and two different $D_g$. **b** The corresponding phase planes of system (12) for $\lambda =0.75$, $c=1$, $D_i=0.25$, $D_g=0.2$ and $D_g=0.6$, respectively. The two solid curves are the nullclines $p=-D(u)R(u)/c$ with $D_g=0.2$ (blue curve) and $D_g=0.6$ (orange curve), respectively. The red dashed lines are the corresponding heteroclinic orbits representing travelling wave solutions in (2) (colour figure online)

### Smooth travelling wave solutions for positive *D*(*U*)

If $D_i<4D_g$, then the nonlinear diffusivity function *D*(*U*) is positive for $U\in [0,1]$, see Fig. 7a. Thus the corresponding system of first-order ODEs (12) is not singular, and the nullcline $p=-D(u)R(u)/c$ does not cross *u*-axis, see Fig. 7b. In other words, (0, 0) and (1, 0) are the only equilibrium points. Following the same method as applied in Sect. 2, we obtain the lower bound

$$\begin{aligned} S_1=\sup _{u\in (0,1)}2\sqrt{\frac{D(u)R(u)}{u}}=\sup _{u\in (0,1)}2\sqrt{\lambda (1-u)D(u)}, \end{aligned}$$

such that there exist smooth monotone travelling wave solutions of (2) for $c\ge S_1$. The origin is still a stable node for $c\ge 2\sqrt{\lambda D_i}:=S_2$ and $S_1\ge S_2$. So, if $S_1\ne S_2$, $c\ge S_1$ is only a sufficient condition because there may exist smooth monotone travelling wave solutions of (2) for wave speeds $S_2\le c< S_1$. Thus, we can only conclude that the minimum wave speed is in the range

38 $$\begin{aligned} S_2\le {\hat{c}}\le S_1, \end{aligned}$$

such that there exist smooth monotone nonnegative travelling wave solutions of (2) for $c\ge {\hat{c}}$. Note that the minimum wave speed ${\hat{c}}$ can be different from the minimum wave speed $c^*$ in Theorem 1, and Lemma 2 does not necessarily hold.

**Fig. 8:**
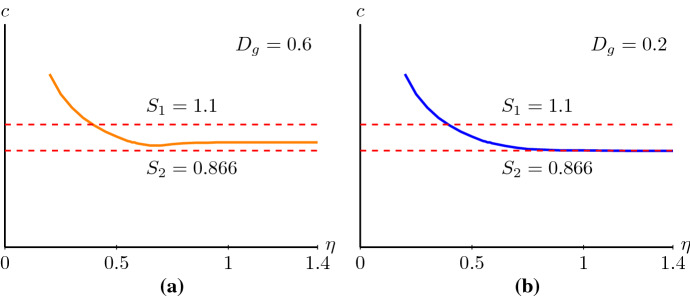
**a** The wave speed as a function of the initial condition $U(x,0)=1/2+\text {tanh}\left( -\eta (x-40)\right) /2$. Notice that as $\eta$ grows to infinity this initial condition limits to the Heaviside initial condition. Parameters are $\lambda =0.75$, $D_i=0.25$ and $D_g=0.6$. The wave speed reaches its minimum which is between $S_1$ and $S_2$ and then converges to a bigger value which is still between $S_1$ and $S_2$. In (**b**), $D_g=0.2$ while the other parameters are the same as in (**a**). In this case, the wave speed converges to $S_2$ (colour figure online)

This estimate is consistent with the result in Malaguti and Marcelli (2003) obtained by using the *comparison method* introduced by Aronson and Weinberger (1978). The corresponding numerical simulations also give the expected results, see Fig. 8. Witelski (1994) obtained an asymptotic travelling wave solution for a PDE motivated by polymer diffusion with a positive nonlinear diffusivity function and logistic kinetics for wave speeds greater than a minimum wave speed which is greater than $S_2$. This is consistent with the estimate of the minimum wave speed in (38). For solutions with an asymptotic wave speed equal to $S_2$, the front of the travelling wave is called a *pulled front*; for solutions with asymptotic speeds greater than $S_2$, the front of the travelling wave is called a *pushed front* (van Saarloos 2003). Unravelling the differences in wave speed selection remains to be explored.

### Shock-fronted travelling waves

In Sect. 2, we mainly considered the equilibrium point (0, 0) as a stable node in the phase plane of system (13). With (0, 0) a stable node, $(\beta ,0)$ is also a stable node based on (22). However, (22) does not hold for any convex *D*(*U*) which changes sign twice. For instance, for

39 $$\begin{aligned} {\hat{D}}(U)=(U-0.1)(U-0.3), \end{aligned}$$

condition (15) and condition (16) become

$$\begin{aligned} c\ge 2\sqrt{{\hat{D}}(0)R'(0)}=0.3,\quad c\ge 2\sqrt{{\hat{D}}'(0.3)R(0.3)}\approx 0.355. \end{aligned}$$

**Fig. 9:**
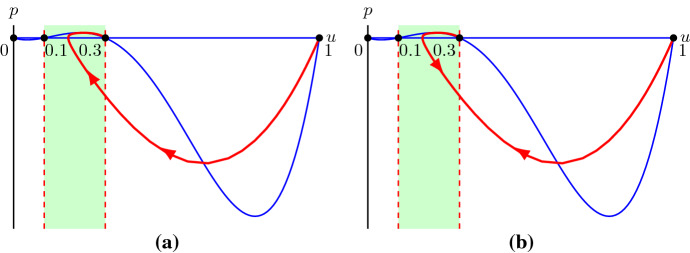
**a** The phase plane of the desingularised system (13) with ${\hat{D}}(u)$, $c=0.3$ and $\lambda =0.75$. The vertical dashed lines are the wall of singularities at $u=0.1$ and $u=0.3$. The blue lines are the nullclines $p=0$ and $p=-D(u)R(u)/c$. The red line is the heteroclinic orbit connecting (1, 0) to (0.3, 0). **b** The phase plane of system (12) with ${\hat{D}}(u)$, $c=0.3$ and $\lambda =0.75$. The vertical dashed lines are the walls of singularities $u=0.1$ and $u=0.3$. The blue lines are the nullclines $p=0$ and $p=-D(u)R(u)/c$. The red line shows the orientation of the same trajectory in (**a**) on different sides of the wall of singularities $u=0.3$ (colour figure online)

**Fig. 10:**
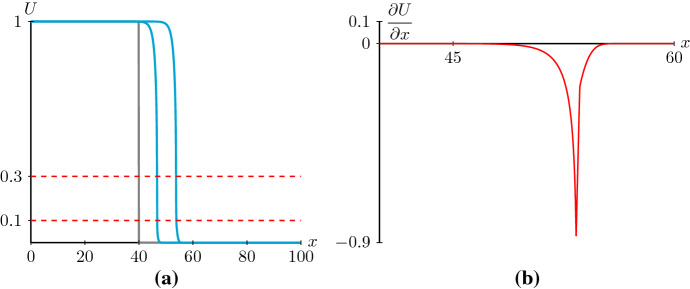
**a** The evolution of a Heaviside initial condition to a shock-fronted travelling wave solution obtained by simulating (2) with (39) and (5) with $\lambda =0.75$ at $t=0$, $t=25$ and $t=50$. Notice that $D(U)=0$ at $\alpha =0.1$ and $\beta =0.3$. The travelling wave solution eventually has a constant positive speed, $c=0.3$. **b** $\partial U/\partial x$ corresponding to the numerical solution in (**a**) at $t=50$ and for *x* between 40 and 60 (colour figure online)

With the nonlinear diffusivity function ${\hat{D}}(U)$, the equilibrium point (0, 0) is a stable node and the equilibrium point $(\beta ,0)$ is a stable spiral for speeds $0.3<c<0.355$ in (13). In this case, only shock-fronted travelling wave solutions of (2) can exist since (13) no longer possesses heteroclinic orbits connecting to $(\beta ,0)$ that do not cross the walls of singularities, see Fig. 9. The corresponding numerical simulation of (2) indeed gives a shock-fronted travelling wave solution with a speed $c=0.3$, see Fig. 10.

It is not a surprise to see shock-fronted travelling wave solutions in negative nonlinear diffusion equations. Shocks in negative nonlinear diffusion equations with no kinetic terms have been studied in the context of many physical phenomena, such as the movement of moisture in partially saturated porous media (DiCarlo et al. 2008); the motion of nanofluids (Landman and White 2011) and these kinds of PDEs also arise in the study of Cahn–Hilliard models (Witelski 1995). Numerical simulations of (2) with nonlinear diffusivity function (3) and Allee kinetics (4) also lead to shock-fronted solutions, see Johnston et al. (2017). In addition, Allee kinetics support shock-fronted travelling wave solutions for reaction–diffusion–advection equations with small diffusion coefficients (Sewalt et al. 2016; Wang et al. 2019). The analysis of shock-fronted travelling wave solutions in nonlinear diffusion–reaction equations with generic diffusivity functions and logistic kinetics is left for future work.

### Point spectrum

The real point spectrum of the operator in (32) is also computable. For this problem we employ the ‘standard’ trick of setting $\theta : = \tan ^{-1}(s/q)$ and then evaluating $d\theta /d\xi$ at where the line (*q*, *s*) is vertical (Jones and Marangell 2012; Harley et al. 2015). In particular, we need to analyse the sign of the following quantity

$$\begin{aligned} \dfrac{d\theta }{d\xi } = \dfrac{-s^2 + \left( \dfrac{D'({\hat{u}})}{D({\hat{u}})} \dfrac{d {\hat{u}}}{d\xi }- c \right) s q + \left( D({\hat{u}})(\varLambda - R'({\hat{u}})) + c \dfrac{D'({\hat{u}})}{D({\hat{u}})} \dfrac{d {\hat{u}}}{d\xi } \right) q^2}{s^2+q^2}\bigg |_{q=0} = 1, \end{aligned}$$

which in particular is independent of $\varLambda$. The implications of this are that if we know the number of times the solution of (32) is vertical for $\varLambda = 0$ as $\xi$ ranges over $\mathbb {R}$ and then again for $\varLambda = \varLambda _{\infty } \gg 1$, then the difference is the number of eigenvalues in the interval $(0,\varLambda _\infty )$ and we can use the previous phase portrait analysis to determine the number of real positive eigenvalues. The number of times the solution of (32) is vertical for $\varLambda =0$ is (as in standard Sturm–Liouville theory) the number of times that the solution curve has a vertical tangent in the phase portrait. This is seen from Fig. 4 as being 0.

## Footnotes

1 Note that there are several other ways, for instance with sub-solutions (Larson 1978), to show that the minimal wave speed for the Fisher–KPP equation is $c^*$.

2 See the introduction of Davis et al. (2017) for definitions, and an explicit computation, of the absolute spectrum for the Fisher–KPP equation.
